# The great automatic grammatizator: On the use and misuse of large language models in scientific and academic writing

**DOI:** 10.1016/j.jdin.2024.10.001

**Published:** 2024-10-16

**Authors:** Jonathan Kantor

**Affiliations:** Department of Dermatology, Center for Global Health, and Center for Clinical Epidemiology and Biostatistics, Perelman School of Medicine at the University of Pennsylvania, Philadelphia, Pennsylvania; Florida Center for Dermatology, St Augustine, Florida

**Keywords:** AI, annotation, artificial intelligence, ChatGPT, Google Gemini, Harry Potter, JK Rowling, large language models, LLM, medical education, Microsoft Bing, peer review, practice management, Roald Dahl

*To the Editor:* Given the excitement surrounding the rise of large language models (LLMs), such as ChatGPT, Google Gemini, and others, it is not surprising that scientific writing is becoming increasingly augmented, or at least influenced, by artificial intelligence.^1^ Seven decades ago, Roald Dahl’s fantastical short story about a frustrated writer trapped in an engineer’s body provided an early look at the potential for artificial intelligence to aid and ultimately supplant human writers; Dahl took particular joy, rather presciently, in highlighting the grammatizator’s singular ability to effortlessly generate consistently mediocre work.^2^

Still, artificial intelligence may serve as a powerful equalizer for those for whom English, the lingua franca of international scientific discourse, is not the first language. Historically (and, indeed, still today), deep-pocketed investigators could rely on professional medical writers to help with the quality of their prose and the structure of their manuscripts; indeed, this practice is particularly common in industry-sponsored research. Given that it is de facto legitimate to use writing help of this sort when one can afford it, why limit the ability of the masses of scientific authors to access similar (if automated) assistance?

If the concern relates to human oversight, then present restrictions—requiring authors to disclose their use of LLMs and, more importantly, to take ultimate responsibility for their output—may be reasonable. However, this touches on a larger, and perhaps more nebulous, question: what are the limits to using artificial intelligence in scientific writing? Using LLMs to help correct style, aid in word choice, and improve transitions is not unreasonable, as this seems to be largely an extension of the ubiquitous spelling and grammar tools that have been used for decades. (As an aside, our comfort with these is likely driven by their integration with word processing software, and as LLMs become increasingly integrated with existing systems, their use will likely seem more natural and expected as well.) Similarly, for qualitative research, it may be reasonable to use LLMs to aid in simple data analysis and coding.^3^

However, as JK Rowling highlighted over 2 decades ago, we should also consider the wisdom of the dictum that one should “never trust anything that can think for itself if you can't see where it keeps its brain.”^4^ Thus, part of the barrier to broader adoption—beyond a sense of unfairness—may also be a (perhaps justifiable) lack of trust. This translates naturally into a visceral sense of discomfort, yielding the increasingly ubiquitous emotional pushback against the use of LLMs.

Indeed, fairness and transparency are important in the writing and peer review process, and most authors would likely appreciate if their work were assessed by a human expert rather than an LLM; similarly, most reviewers would also appreciate if they were not tasked with reviewing work generated in a matter of seconds by an LLM.^5^ However, particularly since we are in a transition period, it is critical to not overcorrect away from using LLMs but instead to maintain flexibility and openness as long as these systems are used as tools rather than ghostwriters.

## Conflicts of interest

None disclosed.
